# Supplementary material for: Surfing motility is a complex adaptation dependent on the stringent stress response in Pseudomonas aeruginosa LESB58
Source: PLoS Pathog. 2020 Mar 24;16(3):e1008444. doi: 10.1371/journal.ppat.1008444 (PMC7122816; doi:10.1371/journal.ppat.1008444)
Supplement: S1 Text — (DOCX) [file ppat.1008444.s001.docx]

**S1 Text.** **The *P. aeruginosa* LESB58 stringent response mutant was impaired in swimming, swarming, adherence, and biofilm formation, as well as being more susceptible to antibiotics**

The LESB58 wild-type strain demonstrated poor in-agar swimming capability [1], cf. surfing motility that appeared normal (Figure 1, S2 Fig). Thus, while swimming motility colonies required almost 48 h to reach the edge of a plate, surfing colonies already reached the edge of the plate within less than 24 h. To explore other stringent response phenotypes, we further investigated swarming motility on semi-solid agar plates, which supported swarming of the LESB58 wild-type strain in a dendritic pattern with pronounced build-ups of bacteria at the end of the dendrites (S1A Fig). In contrast, the Δ*relA*Δ*spoT* mutant, was completely defective in swarming. Complementation with either the *relA* or *spoT* genes restored the ability to migrate, but intriguingly the *relA*-complemented double mutant appeared to have lost its capability to sense its spatial environment and instead of repelling tendrils from adjacent swarming colonies, as is normal for the LESB58 wild-type strain, the two colonies swarmed into each other.

To investigate whether these motility-deficiencies merely reflected poorer growth of the double mutant, we performed growth studies in various media. The Δ*relA*Δ*spoT* mutant had a moderate medium-dependent growth effect in the more nutritionally-deficient growth media KB (S1B Fig) and SCFM (S3A Fig), whereby the growth rate was significantly reduced by 22-38% and the doubling time increased from 80 to 129 min in KB and from 77 to 99 min in SCFM (Table A, B). In contrast, the Δ*relA*Δ*spoT* mutant grew at (>92%) similar rates to the WT in the nutritionally rich complex media Mueller-Hinton Broth and 2xYT. This minor growth defect was complemented with the cloned *spoT* gene but not with the cloned *relA* gene, despite the fact that both genes complemented the swarming defects [NB it is not possible to make a *spoT* knockout since it lacks the ability to degrade ppGpp so we used these chromosomally complemented strains to assess the relative importance of the two genes].

**Table A: Growth rate of *P. aeruginosa* LESB58 strains in different media**. Strains were cultured in a 96-well microtiter plate at 37°C with shaking (567 cpm) and assessed in a microplate reader for 24 h.

| Growth rate ± standard error (h^-1^)^a^ | | | | |
| --- | --- | --- | --- | --- |
| Medium | wild-type | ΔrelAΔspoT | ΔrelAΔspoT/relA^+^ | ΔrelAΔspoT/spoT^+^ |
| KB | 0.52 ± 0.03 | 0.32 ± 0.03^**^ | 0.36 ± 0.01^**^ | 0.54 ± 0.04 |
| dYT | 0.64 ± 0.03 | 0.59 ± 0.04 | 0.56 ± 0.02^*^ | 0.72 ± 0.02 |
| SCFM | 0.54 ± 0.02 | 0.42 ± 0.01^**^ | 0.43 ± 0.03^**^ | 0.54 ± 0.08 |
| MHB | 0.34 ± 0.11 | 0.34 ± 0.12 | 0.21 ± 0.12^*^ | 0.38 ± 0.11 |

^a^ The exponential phase of the growth data was fit with an exponential growth equation, and estimated growth rates were compared with Welch's *t*-test to the wild type. Statistical significance, ^*^ *p* < 0.05, ^**^ *p* < 0.01.

**Table B: Doubling times of *P. aeruginosa* LESB58 strains in different media**. Strains were cultured in a 96-well microtiter plate at 37°C with shaking (567 cpm) and assessed in a microplate reader for 24 h.

| Medium | Doubling time (min) | | | |
| --- | --- | --- | --- | --- |
|  | wild-type | ΔrelA/ΔspoT | ΔrelA/ΔspoT/relA^+^ | ΔrelA/ΔspoT/spoT^+^ |
| KB | 80.04 | 129.30 | 115.26 | 77.10 |
| dYT | 65.10 | 70.26 | 74.88 | 58.03 |
| SCFM | 76.92 | 98.58 | 96.12 | 76.98 |
| MHB | 122.28 | 121.56 | 200.04 | 109.02 |

Since growth cultures showed a strong difference in the coloration of stationary phase cultures for the wild-type compared to the Δ*relA*Δ*spoT* mutant, we tested if this might be due in part to the production of the green-yellow siderophore pyoverdine or blue-pigmented pyocyanin. Indeed, the Δ*relA*Δ*spoT* mutant produced 43% of the amount of pyoverdine (S1E Fig) and 45% of the amount of pyocyanin (S1F Fig) when compared to the wild-type.

Consistent with the inability of other *Pseudomonas* stringent response mutants to form biofilms [3, 4], it was found that the LESB58 Δ*relA*Δ*spoT* mutant was unable to form mature biofilms within three days under flow conditions, and only sparse bacteria, that were often elongated, were observed to be attached to the flow cells (S1D Fig). This might have in part reflected defective adherence since within 1 h in KB, Δ*relA*Δ*spoT* mutant cells adhered only 26.5% as well as wild-type cells, while the *relA* and *spoT* complemented double mutant adhered 75% and 70%, respectively (S1C Fig). Similar results were obtained in dYT and SCFM broth (S4B Fig), which indicated that the prolonged lag phase was not responsible for adherence deficiency.

In addition, antimicrobial susceptibility tests were performed leading to the observation that the Δ*relA*Δ*spoT* mutant had 4- to 8-fold increased susceptibility to multiple antibiotics, including amikacin, tobramycin, aztreonam, cefotaxime, ceftazidime, colistin, erythromycin, and tetracycline (Table C).

**Table C: MIC of LESB58 wild-type, the stringent response double mutant, and complemented strains in MHB.** Changes greater than four-fold are highlighted in bold text.

| **Strain** | **MIC (μg/ml)** | | | | | | | | | | | | |
| --- | --- | --- | --- | --- | --- | --- | --- | --- | --- | --- | --- | --- | --- |
|  | **AMI** | **TOB** | **AZT** | **CTX** | **CAZ** | **MER** | **CIP** | **NFX** | **COL** | **PXB** | **TET** | **AZM** | **ERY** |
| **WT** | 31.3 | 6.3 | 125 | >500 | 31.3 | 3.1 | 3.1 | 12.5 | 3.1 | 6.3 | 31.3 | 62.5 | 250 |
| **Δ*relA*/Δ*spoT*** | **7.8** | **1.6** | **31.3** | **62.5** | **7.8** | 3.1 | 1.6 | 6.3 | **0.8** | 3.1 | **<3.9** | 31.3 | **31.3** |
| **Δ*relA*/Δ*spoT*/*relA*^+^** | 31.3 | 12.5 | 62.5 | >500 | 31.3 | 6.3 | 6.3 | 25 | 3.1 | 12.5 | 31.3 | 62.5 | 500 |
| **Δ*relA*/Δ*spoT*/*spoT*^+^** | 15.6 | 6.3 | 62.5 | >500 | 15.6 | 3.1 | 3.1 | 6.3 | 3.1 | 6.3 | 31.3 | 31.3 | 125 |

AMI, amikacin; AZM, azithromycin; AZT, aztreonam; CTX, cefotaxime; CAZ, ceftazidime; CIP, ciprofloxacin; COL, colistin; ERY, erythromycin; MER, meropenem; NFX, norfloxacin; PXB, polymyxin B; TET, tetracycline; TOB, tobramycin

**References**

1. Kukavica-Ibrulj I, Bragonzi A, Paroni M, Winstanley C, Sanschagrin F, O'Toole GA, et al. In vivo growth of *Pseudomonas aeruginosa* strains PAO1 and PA14 and the hypervirulent strain LESB58 in a rat model of chronic lung infection. J Bacteriol. 2008;190(8):2804-13. doi: 10.1128/JB.01572-07. PubMed PMID: 18083816; PubMed Central PMCID: PMCPMC2293253.

2. Vogt SL, Green C, Stevens KM, Day B, Erickson DL, Woods DE, et al. The stringent response is essential for *Pseudomonas aeruginosa* virulence in the rat lung agar bead and *Drosophila melanogaster* feeding models of infection. Infect Immun. 2011;79(10):4094-104. doi: 10.1128/Iai.00193-11. PubMed PMID: WOS:000294951000026.

3. de la Fuente-Nunez C, Reffuveille F, Haney EF, Straus SK, Hancock REW. Broad-spectrum anti-biofilm peptide that targets a cellular stress response. PLoS Pathog. 2014;10(5). doi: ARTN e1004152, 10.1371/journal.ppat.1004152. PubMed PMID: WOS:000337732300052.

4. Xu X, Yu H, Zhang D, Xiong J, Qiu J, Xin R, et al. Role of ppGpp in *Pseudomonas aeruginosa* acute pulmonary infection and virulence regulation. Microbiol Res. 2016;192:84-95. Epub 2016/09/25. doi: 10.1016/j.micres.2016.06.005. PubMed PMID: 27664726.
