# Supplementary material for: Surfing motility is a complex adaptation dependent on the stringent stress response in Pseudomonas aeruginosa LESB58
Source: PLoS Pathog. 2020 Mar 24;16(3):e1008444. doi: 10.1371/journal.ppat.1008444 (PMC7122816; doi:10.1371/journal.ppat.1008444)
Supplement: S1 Table — Surfing deficiency was defined as either no motility, an alternative form of motility, or one-directional motility. (DOCX) [file ppat.1008444.s002.docx]

**S1 Table.** **PA14 transposon mutants that exhibited surfing deficiency**. Surfing deficiency was defined as either no motility, an alternative form of motility, or one-directional motility.

| **PAO1 Homolog** | **PA14 Locus Tag/Gene Name** | **Description** |
| --- | --- | --- |
| NA | [PA14_03370](http://ausubellab.mgh.harvard.edu/cgi-bin/pa14/view_gene.cgi?GeneID=GID7075) | Hypothetical |
| NA | [PA14_24360](http://ausubellab.mgh.harvard.edu/cgi-bin/pa14/view_gene.cgi?GeneID=GID6249) | Putative serine protease |
| NA | PA14_30910 | Conjugal transfer ATPase TrbE |
| NA | [PA14_46610](http://ausubellab.mgh.harvard.edu/cgi-bin/pa14/view_gene.cgi?GeneID=GID5681) | Putative methyltransferase |
| NA | [PA14_59000](http://ausubellab.mgh.harvard.edu/cgi-bin/pa14/view_gene.cgi?GeneID=GID4176) | Conserved hypothetical |
| NA | PA14_59410 | Hypothetical |
| NA | [PA14_59950](http://ausubellab.mgh.harvard.edu/cgi-bin/pa14/view_gene.cgi?GeneID=GID5242) | Conserved hypothetical |
| PA0026 | *plcB* | Phospholipase C |
| PA0034 | PA14_00430 | Probable two-component response regulator |
| PA0062 | [PA14_00740](http://ausubellab.mgh.harvard.edu/cgi-bin/pa14/view_gene.cgi?GeneID=GID4604) | Putative lipoprotein |
| PA0066 | [*yrdA*](http://ausubellab.mgh.harvard.edu/cgi-bin/pa14/view_gene.cgi?GeneID=GID4103) | Bacterial transferase hexapeptide |
| PA0104 | PA14_01270 | Hypothetical |
| PA0298 | *spuB* | Glutamine synthetase |
| PA0307 | [PA14_04020](http://ausubellab.mgh.harvard.edu/cgi-bin/pa14/view_gene.cgi?GeneID=GID3700) | Conserved hypothetical |
| PA0381 | *thiG* | Thiamine biosynthesis protein, thiazole moiety |
| PA0394 | PA14_05160 | Putative PLP dependent enzyme |
| PA0395 | *pilT* | Twitching motility protein |
| PA0396 | *pilU* | Twitching motility protein |
| PA0406 | [*tonB3*](http://ausubellab.mgh.harvard.edu/cgi-bin/pa14/view_gene.cgi?GeneID=GID2318) | TonB3 involved in iron transport |
| PA0420 | *bioA* | Adenosylmethionine-8-amino-7-oxononanoate aminotransferase |
| PA0428 | *rhlE* | ATP-dependent RNA helicase, DEAD box family |
| PA0429 | [PA14_05580](http://ausubellab.mgh.harvard.edu/cgi-bin/pa14/view_gene.cgi?GeneID=GID1751) | Conserved hypothetical protein |
| PA0462 | PA14_06040 | Hypothetical |
| PA0475 | PA14_06210 | Probable transcriptional regulator |
| PA0501 | *bioF* | 8-amino-7-oxononanoate synthase |
| PA0503 | *bioC* | Putative biotin synthesis protein BioC |
| PA0504 | *bioD* | Dethiobiotin synthase |
| PA0520 | *nirQ* | Denitrification regulatory protein nirQ |
| PA0545 | PA14_07070 | Putative reductase |
| PA0551 | *epd* | D-erythrose 4-phosphate dehydrogenase |
| PA0568 | PA14_07380 | Hypothetical |
| PA0583 | [PA14_07600](http://ausubellab.mgh.harvard.edu/cgi-bin/pa14/view_gene.cgi?GeneID=GID4079) | Putative 2-amino-4-hydroxy-6-hydroxymethyldihydropteridine pyrophosphokinase |
| PA0584 | *cca* | tRNA nucleotidyl transferase |
| PA0594 | *surA* | Peptidyl-prolyl cis-trans isomerase |
| PA0595 | *ostA* | organic solvent tolerance protein ostA |
| PA0624 | [PA14_08100](http://ausubellab.mgh.harvard.edu/cgi-bin/pa14/view_gene.cgi?GeneID=GID4987) | Conserved hypothetical |
| PA0663 | [PA14_08490](http://ausubellab.mgh.harvard.edu/cgi-bin/pa14/view_gene.cgi?GeneID=GID3399) | Conserved hypothetical protein |
| PA0718 | [PA14_48990](http://ausubellab.mgh.harvard.edu/cgi-bin/pa14/view_gene.cgi?GeneID=GID5054) | Hypothetical protein of bacteriophage Pf1 |
| PA0766 | *mucD* | Serine protease mucD precursor |
| PA0773 | *pdxJ* | Pyridoxal phosphate biosynthetic protein |
| PA0794 | PA14_53970 | Aconitate hydratase |
| PA0817 | [PA14_53700](http://ausubellab.mgh.harvard.edu/cgi-bin/pa14/view_gene.cgi?GeneID=GID4545) | Ring-cleaving dioxygenase |
| PA0848 | [*aphB*](http://ausubellab.mgh.harvard.edu/cgi-bin/pa14/view_gene.cgi?GeneID=GID2676) | Alkyl hydroperoxide reductase |
| PA0849 | *trxB2* | Thioredoxin reductase 2 |
| PA0928 | *gacS* | Sensor/response regulator hybrid |
| PA0966 | *ruvA* | Holliday junction DNA helicase |
| PA0973 | [*oprL*](http://ausubellab.mgh.harvard.edu/cgi-bin/pa14/view_gene.cgi?GeneID=GID1381) | Outer membrane lipoprotein |
| PA0974 | PA14_51690 | Conserved hypothetical |
| PA0982 | [*dsbA2*](http://ausubellab.mgh.harvard.edu/cgi-bin/pa14/view_gene.cgi?GeneID=GID3526) | Thioredoxin-like |
| PA0996 | *pqsA* | [Coenzyme A ligase](http://www.pseudomonas.com/feature/show?id=252765) |
| PA0997 | *pqsB* | Homologous to beta-keto-acyl-acyl-carrier protein synthase |
| PA0998 | *pqsC* | Homologous to beta-keto-acyl-acyl-carrier protein synthase |
| PA0999 | *pqsD* | 3-oxoacyl-(acyl carrier protein) synthase III |
| PA1000 | *pqsE* | Quinolone signal response protein |
| PA1003 | *pqsR* | Transcriptional regulator MvfR / PqsR |
| PA1013 | *purC* | Phosphoribosylaminoimidazole-succinocarboxamide synthase |
| PA1033 | *yfcG* | Probable glutathione S-transferase |
| PA1037 | [PA14_50920](http://ausubellab.mgh.harvard.edu/cgi-bin/pa14/view_gene.cgi?GeneID=GID3794) | Conserved hypothetical protein |
| PA1045 | [*dinG*](http://ausubellab.mgh.harvard.edu/cgi-bin/pa14/view_gene.cgi?GeneID=GID272) | ATP-dependent DNA helicase |
| PA1073 | *braD* | Branched-chain amino acid transport protein brad |
| PA1097 | *fleQ* | Transcriptional regulator FleQ; binds cyclic-di-GMP |
| PA1098 | *fleS* | Two-component sensor |
| PA1099 | *fleR* | Transcriptional regulator/response regulator FleR |
| PA1119 | [*yfiB*](http://ausubellab.mgh.harvard.edu/cgi-bin/pa14/view_gene.cgi?GeneID=GID4316) | Prob. outer membrane protein mediates persistence and small colony |
| PA1120 | [*tpbB*](http://ausubellab.mgh.harvard.edu/cgi-bin/pa14/view_gene.cgi?GeneID=GID1324) | Diguanylate cyclase involved in persistence and small colony forming |
| PA1124 | *dgt* | Deoxyguanosinetriphosphate triphosphohydrolase |
| PA1127 | [PA14_49800](http://ausubellab.mgh.harvard.edu/cgi-bin/pa14/view_gene.cgi?GeneID=GID2099) | Probable oxidoreductase |
| PA1157 | PA14_49440 | Probable two-component response regulator |
| PA1187 | [PA14_49080](http://ausubellab.mgh.harvard.edu/cgi-bin/pa14/view_gene.cgi?GeneID=GID1617) | Probable acyl-CoA dehydrogenase |
| PA1210 | [PA14_48650](http://ausubellab.mgh.harvard.edu/cgi-bin/pa14/view_gene.cgi?GeneID=GID3361) | Putative porin protein |
| PA1239 | [PA14_48210](http://ausubellab.mgh.harvard.edu/cgi-bin/pa14/view_gene.cgi?GeneID=GID2293) | Putative hydrolase |
| PA1271 | PA14_47800 | Putative tonB-dependent receptor |
| PA1396 | PA14_46370 | Two-component sensor |
| PA1432 | *lasI* | Autoinducer synthesis protein LasI |
| PA1441 | *fliK* | Putative flagellar hook-length control protein FliK |
| PA1442 | [*fliL*](http://ausubellab.mgh.harvard.edu/cgi-bin/pa14/view_gene.cgi?GeneID=GID4290) | Putative flagellar protein FliL |
| PA1457 | *cheZ* | Chemotaxis protein CheZ |
| PA1463 | PA14_45510 | CheW domain-containing protein |
| PA1483 | *cycH* | Cytochrome c-type biogenesis protein |
| PA1509 | [PA14_44920](http://ausubellab.mgh.harvard.edu/cgi-bin/pa14/view_gene.cgi?GeneID=GID1807) | Conserved hypothetical |
| PA1512 | *hcpA* | Secreted protein Hcp |
| PA1547 | [PA14_44460](http://ausubellab.mgh.harvard.edu/cgi-bin/pa14/view_gene.cgi?GeneID=GID3465) | Putative membrane protein |
| PA1754 | *cysB* | Transcriptional regulator CysB |
| PA1838 | *cysI* | Sulfite reductase |
| PA1935 | [PA14_60080](http://ausubellab.mgh.harvard.edu/cgi-bin/pa14/view_gene.cgi?GeneID=GID394) | Conserved hypothetical |
| PA1982 | *exaA* | PQQ-linked alcohol dehydrogenase |
| PA2002 | [*atoE*](http://ausubellab.mgh.harvard.edu/cgi-bin/pa14/view_gene.cgi?GeneID=GID947) | Short-chain fatty acid transporter |
| PA2009 | *hmgA* | Homogentisate 1,2-dioxygenase |
| PA2089 | PA14_37490 | Putative tonB-dependent receptor |
| PA2120 | [PA14_37150](http://ausubellab.mgh.harvard.edu/cgi-bin/pa14/view_gene.cgi?GeneID=GID4694) | Conserved hypothetical |
| PA2130 | *cupA3* | Usher |
| PA2195 | *hcnC* | Hydrogen cyanide synthase |
| PA2276 | PA14_35140 | AraC family transcriptional regulator |
| PA2524 | *czcS* | Putative two-component sensor |
| PA2576 | PA14_30790 | Putative permease |
| PA2586 | *gacA* | Response regulator GacA |
| PA2587 | *pqsH* | FAD-dependent monooxygenase |
| PA2618 | PA14_30260 | Putative arginyl-tRNA:protein arginylyltransferase |
| PA2685 | PA14_29390 | Conserved hypothetical |
| PA2687 | *pfeS* | Two-component sensor PfeS |
| PA2693 | *vdlD* | Putative long-chain acyl-CoA thioester hydrolase |
| PA2700 | [PA14_29220](http://ausubellab.mgh.harvard.edu/cgi-bin/pa14/view_gene.cgi?GeneID=GID1174) | Putative porin |
| PA2747 | [PA14_28600](http://ausubellab.mgh.harvard.edu/cgi-bin/pa14/view_gene.cgi?GeneID=GID5217) | Conserved hypothetical protein |
| PA2779 | [PA14_28140](http://ausubellab.mgh.harvard.edu/cgi-bin/pa14/view_gene.cgi?GeneID=GID4831) | Hypothetical |
| PA2832 | *tpm* | Thiopurine methyltransferase |
| PA2904 | *cobI* | Precorrin-2 methyltransferase |
| PA2918 | [PA14_26310](http://ausubellab.mgh.harvard.edu/cgi-bin/pa14/view_gene.cgi?GeneID=GID3285) | Putative short-chain dehydrogenase |
| PA2927 | PA14_26190 | Hypothetical proteins |
| PA2936 | [PA14_26070](http://ausubellab.mgh.harvard.edu/cgi-bin/pa14/view_gene.cgi?GeneID=GID3876) | Putative cytochrome b561 |
| PA2969 | *plsX* | Fatty acid/phospholipid synthesis protein |
| PA3050 | *pyrD* | Dihydroorotate dehydrogenase |
| PA3057 | [PA14_24570](http://ausubellab.mgh.harvard.edu/cgi-bin/pa14/view_gene.cgi?GeneID=GID5345) | Hypothetical |
| PA3134 | *gltX* | Putative glutamate-tRNA synthetase |
| PA3197 | PA14_22860 | Putative transcriptional regulator |
| PA3244 | *minD* | Cell division inhibitor MinD |
| PA3280 | *oprO* | Pyrophosphate-specific outer membrane porin |
| PA3324 | [PA14_21050](http://ausubellab.mgh.harvard.edu/cgi-bin/pa14/view_gene.cgi?GeneID=GID489) | Putative short-chain dehydrogenase |
| PA3325 | [PA14_21040](http://ausubellab.mgh.harvard.edu/cgi-bin/pa14/view_gene.cgi?GeneID=GID2395) | Putative hydrolase |
| PA3342 | PA14_20840 | Hypothetical |
| PA3348 | *cheR1* | Chemotaxis protein methyltransferase |
| PA3351 | *flgM* | Flagellin biosynthesis negative regulator FlgM |
| PA3387 | *rhlG* | Beta-ketoacyl reductase |
| PA3472 | [PA14_19170](http://ausubellab.mgh.harvard.edu/cgi-bin/pa14/view_gene.cgi?GeneID=GID3873) | Probable transcriptional regulator |
| PA3476 | *rhlI* | Autoinducer synthesis protein RhlI |
| PA3477 | *rhlR* | Transcriptional regulator RhlR |
| PA3488 | *tli5* | Type 6 secretion system immunity protein |
| PA3489 | PA14_18950 | Putative NADH:ubiquinone oxidoreductase |
| PA3526 | *motY* | Flagella motor |
| PA3546 | *algX* | Alginate biosynthesis protein |
| PA3556 | *arnT* | 4-amino-4-deoxy-L-arabinose lipid A transferase |
| PA3573 | [PA14_18090](http://ausubellab.mgh.harvard.edu/cgi-bin/pa14/view_gene.cgi?GeneID=GID1563) | Putative major facilitator subfamily transporter protein |
| PA3582 | *glpK* | Glycerol kinase |
| PA3599 | PA14_17720 | LuxR family transcriptional regulator |
| PA3628 | [*yeiG*](http://ausubellab.mgh.harvard.edu/cgi-bin/pa14/view_gene.cgi?GeneID=GID2537) | Putative esterase |
| PA3631 | *yedE* | Putative sulphur transport permease protein |
| PA3641 | PA14_17250 | Putative Na+/alanine symporter |
| PA3649 | [*mucP*](http://ausubellab.mgh.harvard.edu/cgi-bin/pa14/view_gene.cgi?GeneID=GID1142) | Membrane-associated zinc metalloprotease regulates alginate |
| PA3697 | PA14_16580 | Hypothetical |
| PA3730 | [PA14_16160](http://ausubellab.mgh.harvard.edu/cgi-bin/pa14/view_gene.cgi?GeneID=GID3782) | Hypothetical |
| PA3735 | *thrC* | Threonine synthase |
| PA3749 | *yhjE* | Probable major facilitator family transporter |
| PA3763 | *purL* | Phosphoribosylformylglycinamidine synthase |
| PA3783 | [PA14_15140](http://ausubellab.mgh.harvard.edu/cgi-bin/pa14/view_gene.cgi?GeneID=GID3821) | Conserved hypothetical |
| PA3818 | *suhB* | extragenic suppressor |
| PA3836 | [PA14_14390](http://ausubellab.mgh.harvard.edu/cgi-bin/pa14/view_gene.cgi?GeneID=GID2324) | Putative ABC-type transport protein |
| PA3858 | PA14_14100 | Putative amino-acid ABC transporter binding protein |
| PA3884 | [PA14_13670](http://ausubellab.mgh.harvard.edu/cgi-bin/pa14/view_gene.cgi?GeneID=GID4818) | Hypothetical |
| PA3892 | PA14_13560 | Putative fusaric acid resistance protein |
| PA3921 | PA14_13150 | Transcriptional regulator |
| PA3948 | *rocA1* | Two-component response regulator RocA1 |
| PA3958 | PA14_12670 | Possible nuclease or phosphotase |
| PA3975 | [*thiD*](http://ausubellab.mgh.harvard.edu/cgi-bin/pa14/view_gene.cgi?GeneID=GID2919) | phosphomethylpyrimidine kinase |
| PA3976 | [*thiE*](http://ausubellab.mgh.harvard.edu/cgi-bin/pa14/view_gene.cgi?GeneID=GID3878) | Possible thiamin-phosphate pyrophosphorylase |
| PA4001 | *sltB1* | Soluble lytic transglycosylase B |
| PA4006 | *nadD* | NadD nicotinic acid mononucleotide adenylyltransferase |
| PA4023 | *eutP* | Putative amino acid transporter |
| PA4050 | *pgpA* | Phosphatidylglycerophosphatase A |
| PA4069 | [PA14_11250](http://ausubellab.mgh.harvard.edu/cgi-bin/pa14/view_gene.cgi?GeneID=GID2507) | Hypothetical protein |
| PA4072 | PA14_11210 | Putative amino acid permease |
| PA4130 | [PA14_10550](http://ausubellab.mgh.harvard.edu/cgi-bin/pa14/view_gene.cgi?GeneID=GID574) | Putative sulfite or nitrite reductase |
| PA4137 | PA14_10440 | Putative porin |
| PA4144 | [PA14_10330](http://ausubellab.mgh.harvard.edu/cgi-bin/pa14/view_gene.cgi?GeneID=GID2843) | Putative outer membrane protein precursor |
| PA4168 | [*fpvB*](http://ausubellab.mgh.harvard.edu/cgi-bin/pa14/view_gene.cgi?GeneID=GID168) | Type I ferripyoverdine receptor, FpvB |
| PA4208 | *opmD* | Outer membrane protein |
| PA4210 | *phzA1* | Probable phenazine biosynthesis protein |
| PA4233 | [PA14_09190](http://ausubellab.mgh.harvard.edu/cgi-bin/pa14/view_gene.cgi?GeneID=GID6612) | Putative MFS transporter |
| PA4332 | *sadC* | diguanylate cyclase involved in biofilm formation |
| PA4333 | *fumA* | Putative fumarate hydrolase |
| PA4398 | PA14_57170 | Two-component sensor involved in swarming and biofilm formation |
| PA4431 | PA14_57570 | Putative cytochrome c reductase, iron-sulfur subunit |
| PA4455 | PA14_57870 | Putative toluene tolerance ABC efflux transporter |
| PA4462 | *rpoN* | RNA polymerase factor sigma-54 |
| PA4471 | *fagA* | Hypothetical |
| PA4511 | [PA14_58540](http://ausubellab.mgh.harvard.edu/cgi-bin/pa14/view_gene.cgi?GeneID=GID3156) | Conserved hypothetical protein |
| PA4518 | [PA14_58620](http://ausubellab.mgh.harvard.edu/cgi-bin/pa14/view_gene.cgi?GeneID=GID4579) | Conserved hypothetical |
| PA4552 | *pilW* | Type 4 fimbrial biogenesis protein |
| PA4566 | *obg* | GTP-binding protein, GTP1/Obg family |
| PA4612 | [PA14_61020](http://ausubellab.mgh.harvard.edu/cgi-bin/pa14/view_gene.cgi?GeneID=GID4129) | Ankyrin-like protein |
| PA4616 | PA14_61080 | Probable C4-dicarboxylate-binding protein |
| PA4650 | PA14_61520 | Conserved hypothetical |
| PA4695 | *ilvH* | Acetolactate synthase isozyme III small subunit |
| PA4725 | *cbrA* | Two-component sensor CbrA |
| PA4726 | *cbrB* | Two-component response regulator CbrB |
| PA4729 | *panB* | 3-methyl-2-oxobutanoate hydroxymethyltransferase |
| PA4734 | [PA14_62640](http://ausubellab.mgh.harvard.edu/cgi-bin/pa14/view_gene.cgi?GeneID=GID2580) | Conserved hypothetical protein |
| PA4743 | *rbfA* | Ribosome-binding factor A |
| PA4752 | *rrmJ* | Cell division protein |
| PA4753 | [*yhbY*](http://ausubellab.mgh.harvard.edu/cgi-bin/pa14/view_gene.cgi?GeneID=GID5137) | Putative RNA-binding protein |
| PA4758 | *carA* | Carbamoyl-phosphate synthase small chain |
| PA4778 | *cueR* | Cu(I)-responsive transcriptional regulator CueR |
| PA4831 | PA14_63880 | Probable transcriptional regulator |
| PA4838 | [PA14_63970](http://ausubellab.mgh.harvard.edu/cgi-bin/pa14/view_gene.cgi?GeneID=GID1538) | Putative membrane protein |
| PA4854 | *purH* | Phosphoribosylaminoimidazolecarboxamide transferase |
| PA4855 | *purD* | Phosphoribosylamine--glycine ligase |
| PA4930 | *alr* | Biosynthetic alanine racemase |
| PA4953 | *motB* | Chemotaxis protein MotB |
| PA4959 | *fimX* | Phosphodiesterase involved in twitching motility; polar localization. |
| PA4975 | [PA14_65760](http://ausubellab.mgh.harvard.edu/cgi-bin/pa14/view_gene.cgi?GeneID=GID3341) | NAD(P)H quinone oxidoreductase |
| PA4981 | [PA14_65850](http://ausubellab.mgh.harvard.edu/cgi-bin/pa14/view_gene.cgi?GeneID=GID969) | Putative amino acid ABC transporter, permease protein |
| PA5015 | *aceA* | Pyruvate dehydrogenase, E1 component |
| PA5017 | *dipA* | Phosphodiesterase involved in biofilm dispersion, virulence, persistence |
| PA5025 | *metY* | Homocysteine synthase |
| PA5070 | *tatC* | Sec-independent protein translocase |
| PA5076 | [PA14_67050](http://ausubellab.mgh.harvard.edu/cgi-bin/pa14/view_gene.cgi?GeneID=GID2972) | ABC transporter, periplasmic amino acid (glutamate)-binding protein |
| PA5109 | [PA14_67470](http://ausubellab.mgh.harvard.edu/cgi-bin/pa14/view_gene.cgi?GeneID=GID3760) | Conserved hypothetical |
| PA5174 | [PA14_68360](http://ausubellab.mgh.harvard.edu/cgi-bin/pa14/view_gene.cgi?GeneID=GID427) | Putative beta-ketoacyl synthase |
| PA5192 | *pckA* | Phosphoenolpyruvate carboxykinase |
| PA5193 | *hslO* | Putative chaperon |
| PA5203 | *gshA* | Glutamate--cysteine ligase |
| PA5277 | *lysA* | Diaminopimelate decarboxylase |
| PA5323 | *argB* | Acetylglutamate kinase |
| PA5331 | *pyrE* | Orotate phosphoribosyltransferase |
| PA5368 | *pstC* | Phosphate ABC transporter, permease protein |
| PA5376 | *cbcV* | Choline/betaine/carnitine ABC transporter, ATP-binding subunit |
| PA5399 | [*dgcB*](http://ausubellab.mgh.harvard.edu/cgi-bin/pa14/view_gene.cgi?GeneID=GID338) | Dimethyl glycine metabolism |
| PA5454 | *rmd* | Oxidoreductase |
| PA5555 | *atpG* | ATP synthase gamma chain |
| PA5561 | *atpI* | ATP synthase protein I |
