# Supplementary material for: Surfing motility is a complex adaptation dependent on the stringent stress response in Pseudomonas aeruginosa LESB58
Source: PLoS Pathog. 2020 Mar 24;16(3):e1008444. doi: 10.1371/journal.ppat.1008444 (PMC7122816; doi:10.1371/journal.ppat.1008444)
Supplement: S2 Table — (DOCX) [file ppat.1008444.s003.docx]

**S2 Table: Primers used in this study.**

| **Primer** | **Sequence (5' - 3')** |
| --- | --- |
| PA0034_qF | CAGGGAATGGGCGTTGAGTT |
| PA0034_qR | CAACACCAACAAGGCCATCG |
| cheY_qF | CATCAAGAACCTCTTGCGGG |
| cheY_qR | AATTGCCGCTGTGCAGCATC |
| nirQ_qF | CGGCCATGAGATCGAAGTCT |
| nirQ_qR | TACTGGACGAAGCGGGTCTT |
| pqsR_qF | CATGTTCCTCCAGGTCATCG |
| pqsR_qR | GATTTCCAGGTTGCTGACCG |
| fleQ_qF | GTAGGGATGCATGATCGCCA |
| fleQ_qR | TTCAACTCGGCGGCGATCAT |
| fleR_qF | CCTGAATCGCAGAAAGAGGC |
| fleR_qR | CAATTGCAGTTGCGCTCTCG |
| lasR_qF | TCACATTGGCTTCCGAGCAG |
| lasR_qR | AAACCGGTGGTTCTGACCAG |
| pqsH_qF | CGAATTCACCAAGGCAGGCA |
| pqsH_qR | AACGCCGTCAACGCATTGCT |
| rhlR_qF | GTCCATGGCACCTATCCCAA |
| rhlR_qR | AGACCACCATTTCCGAGGAG |
| rhlB_qF | CCTCATCTCGATCATCACCC |
| rhlB_qR | ATCTGTACCACCAGTTCGCG |
| PA3599_qF | CGCTGGTAGTAATCGGTCTG |
| PA3599_qR | TACCTGCTCGACCCGTTCTA |
| PA3921_qF | AAGAGTTCCCTGGCCATCGA |
| PA3921_qR | TTCGAGGAAACGTCCGGGAT |
| PA4398_qF | CTCAACGCGCTGATCGAACT |
| PA4398_qR | TGAGGGTTCCGGTGTAGAAC |
| cbrB_qF | ATCCGATCTGCATTGCGACG |
| cbrB_qR | ACCATGTCGAAGGAGGGGAT |
| cueR_qF | GCGAAGAAAAGCGGACTGAC |
| cueR_qR | TCGTTCTCGTTGTAGTGGCG |
| cueR_oe_fwd-Kpn | CTGGGTACCGCGGGCGGTCACATATCTGA |
| cueR_oe_rev-Hin | GATAAGCTTGGATATCAGTGGCAGCAGCC |
| pqsH_oe_fwd-Apa | GGGGGGCCCGGTTACCTCTTGCACGCGA |
| pqsH_oe_rev-Hin | GATAAGCTTAGCGACCATCGCCGAAGTC |
