# Supplementary figures and images for: Surfing motility is a complex adaptation dependent on the stringent stress response in Pseudomonas aeruginosa LESB58
Source: PLoS Pathog. 2020 Mar 24;16(3):e1008444. doi: 10.1371/journal.ppat.1008444 (PMC7122816; doi:10.1371/journal.ppat.1008444)

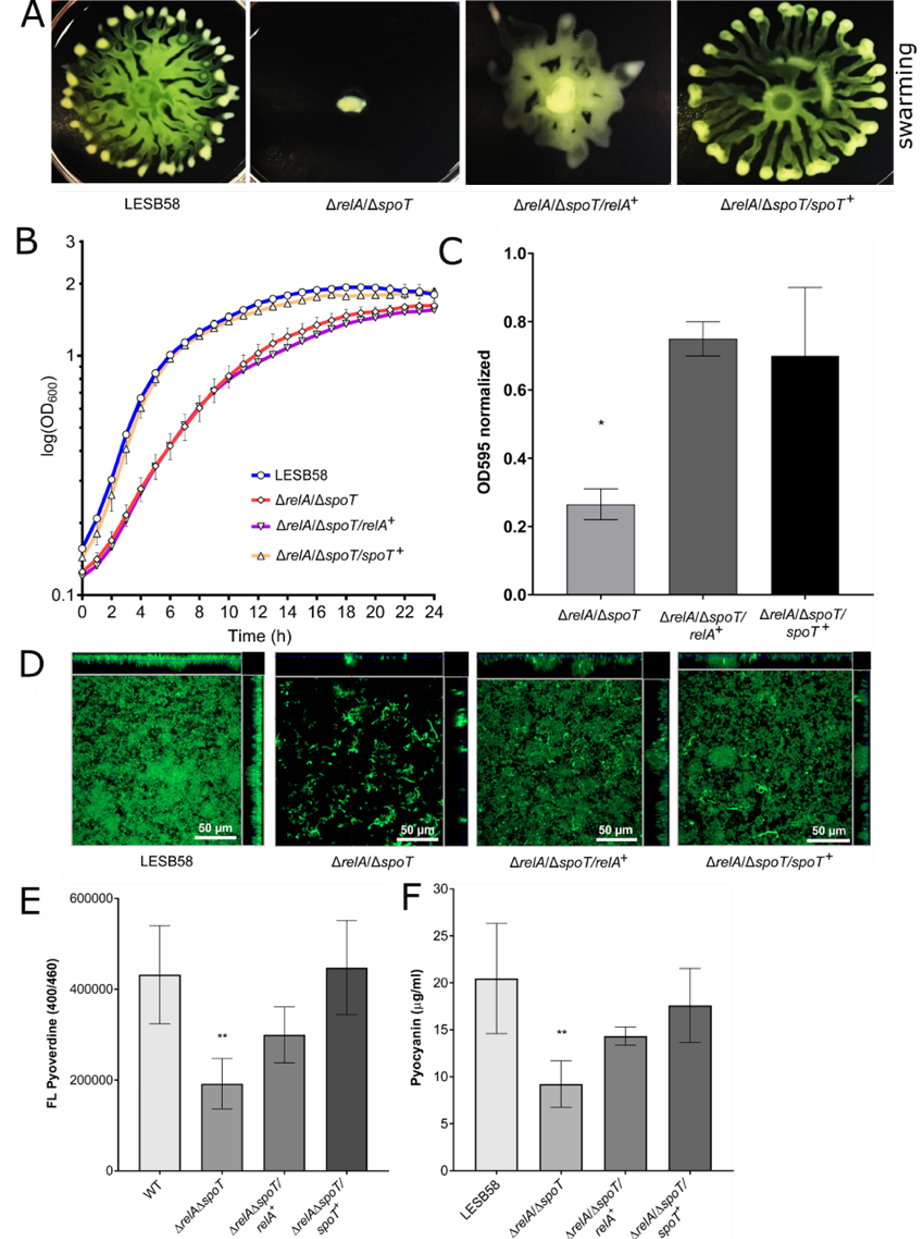

Supplement: S1 Fig — A) Swarming on plates with 0.4% agar in KB medium. Plates were incubated for 48 h at 37°C. Experiments were repeated at least three times with similar results. B) Bacterial growth in KB liquid broth in a 96-well microtiter plate reader at 37°C under shaking conditions (567 cpm) for 24 h. C) One-hour adherence to plastic in KB medium at room temperature. OD values were normalized to the wild-type absorbance. * indicates p-value < 0.05 compared to wild-type. Experiments were performed at least three times. Error bars indicate ± standard error. D) Biofilm formation under flow-cell conditions in dYT broth. Cells were stained after three days for one hour with 1 μM SYTO-9 and subsequently imaged using a Zeiss LSM800 confocal microscope. E) Pyoverdine production (fluorescence: excitation 400 nm; emission 460 nm) after 20 h incubation in KB broth in a 96-well plate under shaking conditions (567 cpm). F) Pyocyanin production after 22 h incubation in SCFM broth under shaking conditions (220 rpm). E, F) Analysis was performed using One-way ANOVA with Dunn correction. **, indicate p-value < 0.01 compared to wild-type. D-F) Experiments were performed 2–3 times. Error bars indicate ± standard deviation. (PNG) [file ppat.1008444.s004.png]

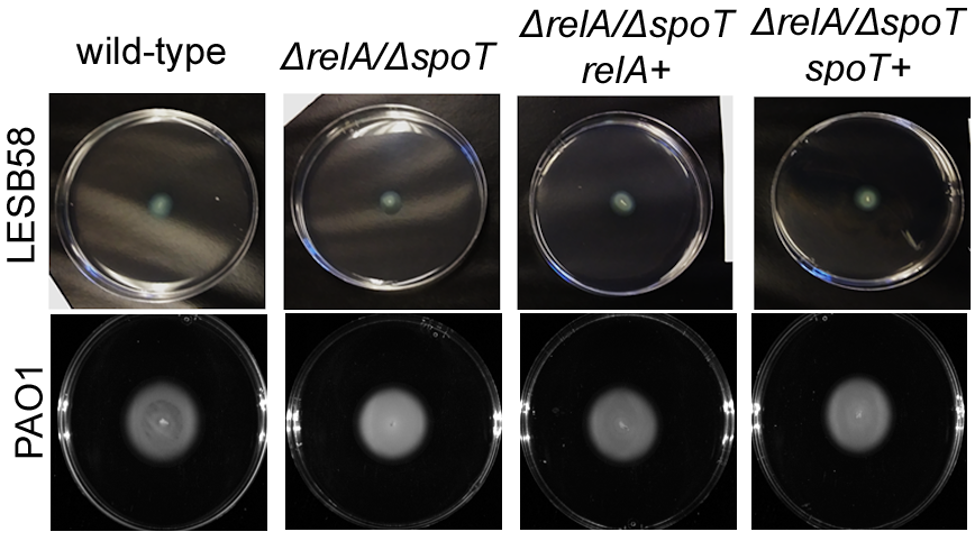

Supplement: S2 Fig — Stringent response mutants and complements were tested under swimming conditions in KB 0.3% agar plates for 24 h (LESB58, top) and SCFM 0.3% agar plates for 15 h (PAO1, bottom) at 37°C. (PNG) [file ppat.1008444.s005.png]

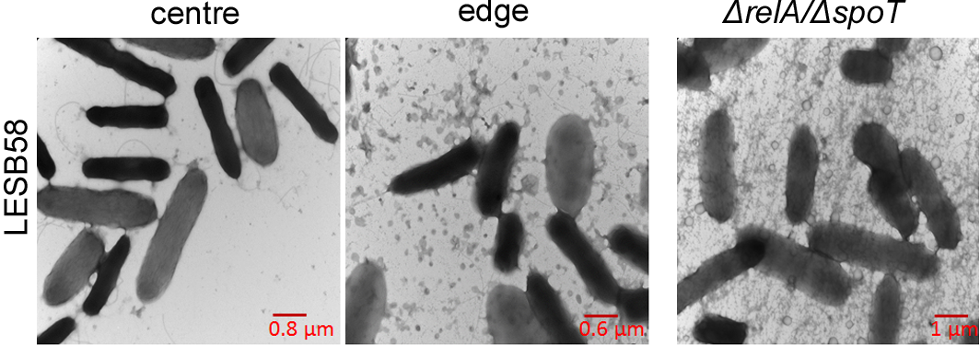

Supplement: S3 Fig — Representative images taken from the centre (left) and edge (middle) of a surfing (SCFM supplemented with 0.4% mucin agar plate) colony, and stringent response mutant (right). Experiments were repeated three times with similar results. (PNG) [file ppat.1008444.s006.png]

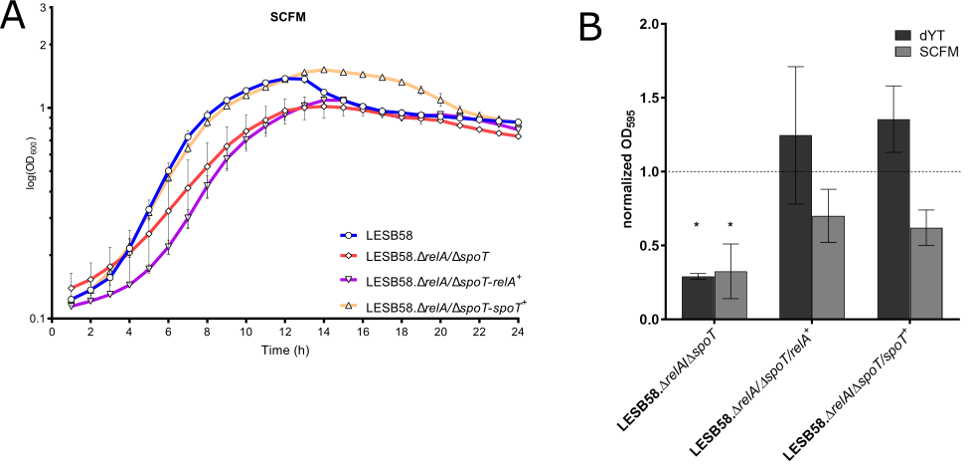

Supplement: S4 Fig — A) Bacterial growth in SCFM liquid broth in a 96-well microtiter plate reader at 37°C under shaking conditions (567 cpm) for 24 h. B) One-hour adherence to plastic in dYT and SCFM medium at room temperature. OD values were normalized to the wild-type absorbance. * indicates p-value < 0.05 compared to wild-type. Experiments were performed at least three times. Error bars indicate ± standard error. (PNG) [file ppat.1008444.s007.png]

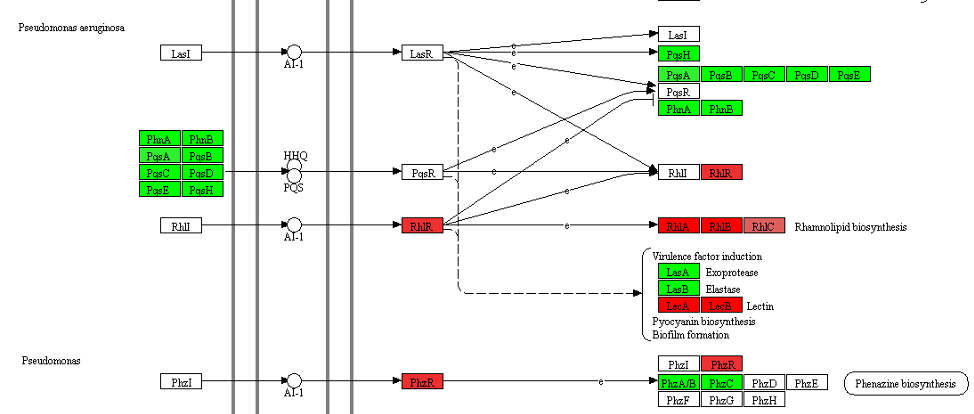

Supplement: S5 Fig — Visualization of DE genes of the stringent response mutant vs. wild-type under surfing conditions. The quorum sensing pathway (pae02024) was visualized using Pathview. Green boxes indicate a downregulation; red boxes upregulation. (PNG) [file ppat.1008444.s008.png]

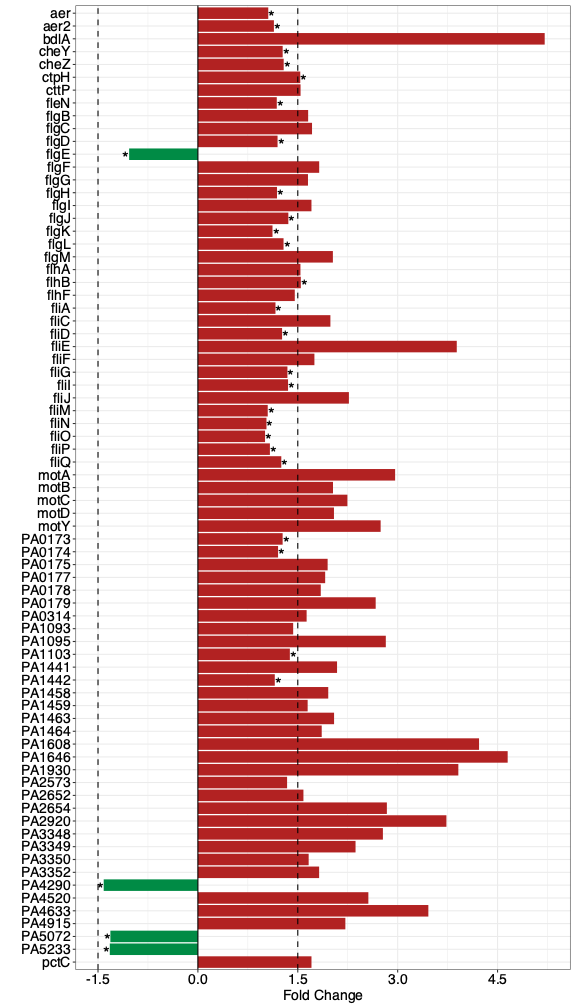

Supplement: S6 Fig — Visualization of DE genes of the stringent response mutant vs. wild-type under surfing conditions. Red bar plots indicate upregulation and green bars downregulation. The asterisks indicate genes with low confidence (adjusted p-value > 0.05). Dashed line shows significance threshold based on fold change. Gene list was downloaded from KEGG. (PNG) [file ppat.1008444.s009.png]

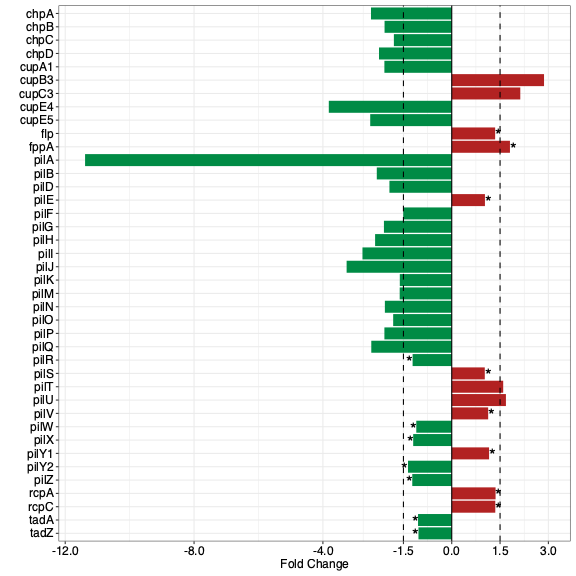

Supplement: S7 Fig — Visualization of DE genes of the stringent response mutant vs. wild-type under surfing conditions. Red bar plots indicate upregulation and green bars downregulation. The asterisks indicate genes with low confidence (adjusted p-value > 0.05). Dashed line shows significance threshold based on fold change. Gene list was downloaded from KEGG. (PNG) [file ppat.1008444.s010.png]

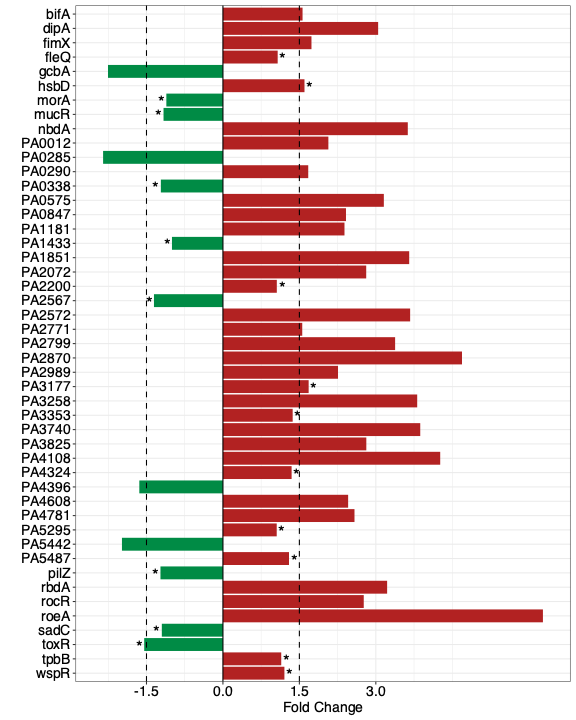

Supplement: S8 Fig — Visualization of DE genes of the stringent response mutant vs. wild-type under surfing conditions. Red bar plots indicate upregulation and green bars downregulation. The asterisks indicate genes with low confidence (adjusted p-value > 0.05). Dashed line shows significance threshold based on fold change. Gene list was downloaded from https://www.ncbi.nlm.nih.gov/Complete_Genomes/c-di-GMP.html. (PNG) [file ppat.1008444.s011.png]

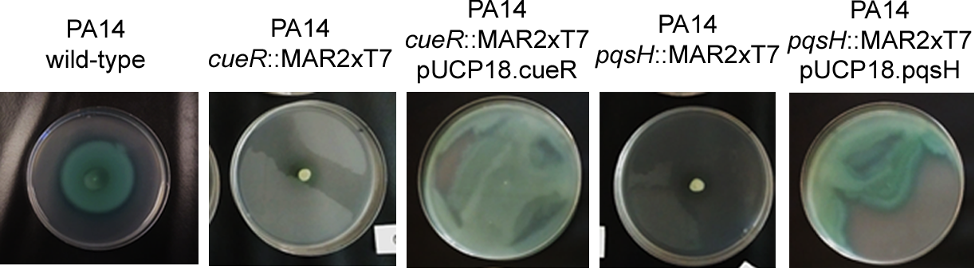

Supplement: S9 Fig — Surfing on 0.3% SCFM agar supplemented with 0.4% mucin. All strains were grown for 16–18 h at 37°C and experiments were repeated at least 3 times with similar results. (PNG) [file ppat.1008444.s012.png]

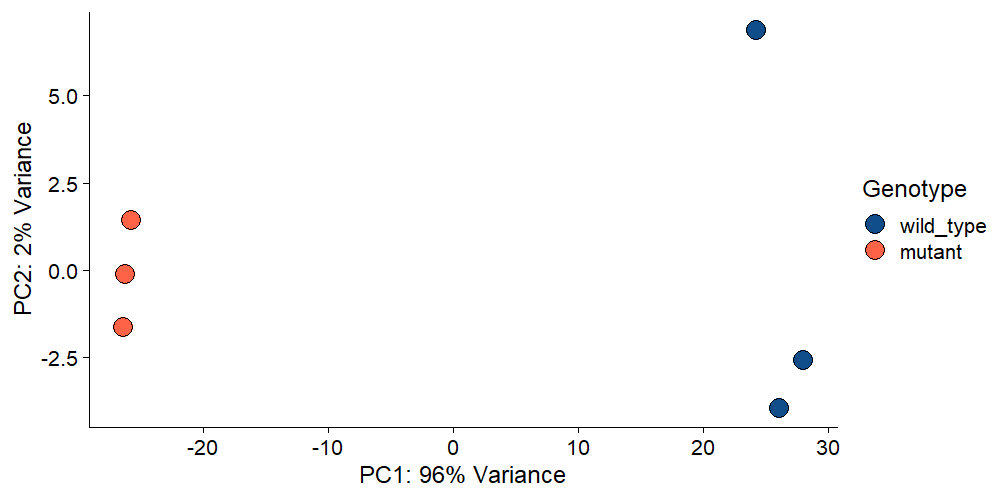

Supplement: S10 Fig — (PNG) [file ppat.1008444.s013.png]
