# Supplementary material for: Surfing motility is a complex adaptation dependent on the stringent stress response in Pseudomonas aeruginosa LESB58
Source: PLoS Pathog. 2020 Mar 24;16(3):e1008444. doi: 10.1371/journal.ppat.1008444 (PMC7122816; doi:10.1371/journal.ppat.1008444)
Supplement: Attachment — Submitted filename: Pletzer_PlosPathogens_2020_response2reviewers.docx [file ppat.1008444.s015.docx]

Dear Editor,

Attached please find our response to the reviewer’s enquiries. We have responded in plain type for each issue in turn with the reviewers comments in italics. Modifications into the manuscript text and supplementary information are red in the marked copy of the manuscript and underlined in the responses to the reviewer’s comments. The indicated line numbers refer to the unmarked manuscript version.

Sincerely Yours

R.E.W. Hancock,

UBC Killam Professor and Canada Research Chair

*Responses to Questions*

**Part I – Summary**

***Reviewer #1:*** *The paper by Pletzer et al entitled " Surfing motility is a complex adaptation that is different from swarming motility and requires the stringent stress response in PA LESB58"
This is a well written paper that demonstrates that mutating the genes required for the stringent response in PA has multiple phenotypes for LESB58 related to surface behaviors. The authors then further explore surfing motility with transcriptional profiling of wt and stringent response mutants as well as transposon mutagenesis to identify genes required for surfing and that these have little overlap with swarming genes. In both of these experiments a large data set is generated and the authors complement cueR and pqsR specifically to show these are down-stream regulators of the stringent response that are required for surfing.*

*Strengths: Large data set generated with sound experimental design. The role of the stringent response in surfing is clear and further evidence is presented to differentiate surfing and swarming. The significance is that it offers new insight into the role of the stringent response in surface behaviors.*

*Opportunities: This is primarily a descriptive study with large amounts of genetic data and there is an opportunity as described below to better understand mechanism as well as how the different pieces of data connect. Some of the data such as the antibiotic susceptibility testing doesn't seem to fit with the rest of the manuscript and as an example no mechanism with regard to the antibiotic susceptibility is explained from additional transcriptional data or even discussed. Additional control experiments will help understand how much of the transcriptional change is specific to the stringent response defect compared with actual surfing.*

We thank the reviewer for this feedback. In response to reviewer #2, we further streamlined the manuscript to specifically focus on surfing motility and have moved other phenotypes that distract from the main story into Supplementary. Regarding additional control experiments, we previously performed RNA-Seq (surf vs. swim) to determine the surfing regulon in PA14 [1] in synthetic cystic fibrosis sputum media (SCFM). Due to the different experimental conditions of the previous study and the current one (LESB vs PA14, KB medium vs SCFM) we did not perform a direct comparison here. We appreciate the comment and agree that it would be of interest to perform this transcriptomic comparison in the future under the same experimental conditions.

1. Sun E, Gill EE, Falsafi R, Yeung A, Liu S, Hancock REW. Broad-spectrum adaptive antibiotic resistance associated with *Pseudomonas aeruginosa* mucin-dependent surfing motility. Antimicrob Agents Chemother. 2018;62(9). Epub 2018/07/04. doi: 10.1128/AAC.00848-18. PubMed PMID: 29967020.

***Reviewer #2:*** *The study reports on numerous aspects of a clinical isolate of Pseudomonas aeruginosa, strain LESB58, a previously identified CF lung isolate. Primarily, the manuscript reports upon phenotypes for a double- mutant of relA and spoT, where the stringent response is eliminated, are compared to WT and complemented strains. This research is very useful to elucidate how stringent response controls P. aeruginosa behavior.*

*The short title is a more fitting description than the full manuscript title. However, the current version of the manuscript requires some work to establish a singular theme.*

We have moved the distracting parts from the main manuscript to the Supplementary section to further focus only on surfing motility and the stringent stress response. We have updated the short title to: “Surfing motility is stringently controlled in *Pseudomonas aeruginosa*”. Regarding the full title we changed it to “Surfing motility is a complex adaptation dependent on the stringent stress response in *Pseudomonas aeruginosa* LESB58”

***Reviewer #3:*** *The manuscript entitled: Surfing motility is a complex adaptation that is different from swarming motility and requires the stringent stress response in P. aeruginosa, is an original and well written manuscript with well-planned experiments, careful analysis and novel data on invasion and virulence important in CF. The authors have used a systematic and well-described approach initially starting with phenotypic studies, transcriptome profiling with the LESB58 so-called hyper virulent strain and then moving on to the prototype PA14 strain having a wide pathogenicity profile, and finally qRT-PCR. In a classic analysis and as expected, the authors have used deletion mutants to support their initial observations that the stringent response may be involved. Also, these mutants were found to be susceptible to several antibiotics. Very well done and excellent data, overall.*

*One of the challenges of this type of study is to delineate clearly the surfing phenotype from swarming, twitching and swimming. The authors have shown this previously in different bacterial species using elegant strategies (J. Bacteriol., 2018). Here, the key challenge is the transcriptomics data.*

*One important item I may have missed: Were the samples done in at least triplicates in the RNASeq analysis? The authors have to address this. I think the authors have to discuss the ramifications of the impact of 2 to 7.7 fold changes up or down in gene expression when doing transcriptomics in bacteria. It would be an opportunity to educate some of the readers that fold changes in bacteria remains a challenge. This is on page 9 line 2019 and applies even with qRT-PCR.*

Thank you. We have addressed these comments below. The RNA-Seq study was done in triplicate (i.e. as 3 independent biological repeats).

***Part II – Major Issues: Key Experiments Required for Acceptance***

*Please use this section to detail the key new experiments or modifications of existing experiments that should be absolutely required to validate study conclusions.*

***Reviewer #1:*** *There are experiments and analysis that would significantly strengthen the manuscript.
1) Most of the phenotypes attributed to the stringent response mutants can be attributed to loss or decrease of flagella function and possibly abnormal pilus function. Do these structures change on the cell surface in number or location?*

There is no basic loss of flagella due to these mutations as judged by swimming motility after 24 h; so if there is any defect it must be specific to swarming and surfing cells. To address this, we performed transmission electron microscopy (TEM) on cells isolated from wild-type surfing cells as well as stringent response non-surfers. However, the determination and quantification of the presence of flagella at the surface of cells taken from agar plates as done here is very challenging using TEM analysis. Flagella might break off during the preparation procedure and the poor resolution of the structure itself limits interpretation. Another issue, as we have shown previously, is that the loss of flagella in cells at the thick outer edge of surfing colonies is an intrinsic property of this type of motility and one that strongly differentiates it from swarming. Due to these limitations and potential misinterpretation of the results, we therefore did not specifically focus on these kinds of experiments to verify mechanisms.

We have added a corresponding set of results including TEM data to Supplementary:

Ln112-125: “There was no basic defect in flagella production due to any of these mutations, since we observed similar swimming zone sizes after 24 h for WT and all mutants in both the strain LESB58 and PAO1 backgrounds (although the swimming colony morphology for strain LESB58 was somewhat unusual). Furthermore, all mutants appeared to show similar swimming ability when viewed in aqueous suspension using a light microscope. Nevertheless, to investigate if the loss of surfing involved flagella loss, we examined cell morphology of mutant cells on surfing agar plates and compared them to wild-type cells using transmission electron microscopy (TEM). The LESB58 wild-type showed two distinct morphologies with electron-dense thin rods and less dense enlarged cells at the edge and center, while the stringent response mutant only exhibited enlarged cells that were both longer and wider than wild-type cells (Figure S4). Flagella could only be observed on cells at the centre of surfing plates, but not at the edge for WT, as previously observed [11]. We were unable to detect flagella in the spots formed by the Δ*relA*Δ*spoT* double mutant (Figure S4), although the large amount of extracellular material under these circumstances made observations difficult.”

*Are there more flagella that don’t work as well to explain the upregulation of the fle genes?*

We did not observe hyper-flagellated cells in this or any previous studies although hyperflagellation has been shown to be a feature of swarming cells in *Pseudomonas*. Again, there are limitations in performing electron microscopy from cells taken from plates with low agar concentrations and the potential for flagella that were washed off during the preparation. Hyper-flagellated cells could indeed explain the reduction in motility as shown by others [1, 2].

We have added the following sentence to the discussion:

Ln234-236: “While we did not observe more flagella on the stringent response mutant cells in TEM, a possible explanation could be that the upregulation of the *fle* genes could lead to hyper-flagellated cells that might impair motility [1, 2].”

1. Konishi M, Kanbe M, McMurry JL, Aizawa S. Flagellar formation in C-ring-defective mutants by overproduction of FliI, the ATPase specific for flagellar type III secretion. J Bacteriol. 2009;191(19):6186-91. Epub 2009/08/04. doi: 10.1128/JB.00601-09. PubMed PMID: 19648242; PubMed Central PMCID: PMCPMC2747906.
2. Schuhmacher JS, Thormann KM, Bange G. How bacteria maintain location and number of flagella? FEMS Microbiol Rev. 2015;39(6):812-22. Epub 2015/07/22. doi: 10.1093/femsre/fuv034. PubMed PMID: 26195616.

*Is twitching motility intact?*

We have not investigated twitching motility of LESB58 in this study, since surfing does not involve pili, although we confirmed reduced surface associated adherence which is also a pilus-associated property. The work by Kukavica-Ibrulj et al [1] and Jeukens et al [2] showed that LESB58 has the ability to twitch, albeit slower than other reference strains such as PAO1. Vogt et at [3] demonstrated the involvement of the stringent response in twitching in the reference strain PAO1.

1. Kukavica-Ibrulj I, Bragonzi A, Paroni M, Winstanley C, Sanschagrin F, O'Toole GA, et al. In vivo growth of *Pseudomonas aeruginosa* strains PAO1 and PA14 and the hypervirulent strain LESB58 in a rat model of chronic lung infection. J Bacteriol. 2008;190(8):2804-13. doi: 10.1128/JB.01572-07.
2. Jeukens J, Boyle B, Kukavica-Ibrulj I, Ouellet MM, Aaron SD, Charette SJ, et al. Comparative genomics of isolates of a *Pseudomonas aeruginosa* epidemic strain associated with chronic lung infections of cystic fibrosis patients. PLoS One. 2014;9(2):e87611. Epub 2014/02/08. doi: 10.1371/journal.pone.0087611.
3. Vogt SL, Green C, Stevens KM, Day B, Erickson DL, Woods DE, et al. The stringent response is essential for *Pseudomonas aeruginosa* virulence in the rat lung agar bead and *Drosophila melanogaster* feeding models of infection. Infect Immun. 2011;79(10):4094-104. doi: 10.1128/Iai.00193-11.

*Are there phenotypic changes in rhamnolipid or exopolysaccharide production that can explain any of these surface changes,*

Surfing is independent of rhamnolipids production (unlike swarming) [1]. Schafhauser et al [2] showed that stringent response mutants were impaired in rhamnolipid production in PAO1. We have performed similar experiments, but under the growth conditions tested, the LESB58 wild-type did not produce sufficient rhamnolipids for reliable measurements. Intriguingly, rhamnolipid production as well as quorum sensing regulators are upregulated in the stringent response mutant under surfing conditions. We have added this new data in Table 1 as described below.

1. Yeung AT, Parayno A, Hancock REW. Mucin promotes rapid surface motility in *Pseudomonas aeruginosa*. MBio. 2012;3(3). doi: 10.1128/mBio.00073-12.
2. Schafhauser J, Lepine F, McKay G, Ahlgren HG, Khakimova M, Nguyen D. The stringent response modulates 4-hydroxy-2-alkylquinoline biosynthesis and quorum-sensing hierarchy in *Pseudomonas aeruginosa*. J Bacteriol. 2014;196(9):1641-50. doi: 10.1128/JB.01086-13. PubMed PMID: 24509318; PubMed Central PMCID: PMCPMC3993315.

*If one looks at the cells under the microscope with increasing viscosity what happens to swimming.*

We are uncertain as to why this question is being asked. We and others have previously shown that increasing viscosity (through increases of agar concentrations, causes a switch from in agar swimming to surface motility in the absence (swarming) or presence (surfing) of mucin. When we investigated planktonic cells under the light microscope, the stringent response mutant cells were swimming under a coverslip in a similar coordinated manner as the wild-type, with no difference between the two strains. With increased viscosity in glycerol, we observed slower motility, but still no obvious motility difference between the wild-type and stringent response mutant. This is not the same as surfing on the surface of an agar plate however.

We have added the following statement:

Ln112-115: ”There was no basic defect in flagella production due to any of these mutations, since we observed similar swimming zone sizes after 24 h for WT and all mutants in both the strain LESB58 and PAO1 backgrounds (although the swimming colony morphology for strain LESB58 was somewhat unusual).”

*Phenotypic data (such as transmission electron microscopy) would begin to suggest a mechanism for the changes and also when the CueR and PqsH are overexpressed, to correct surfing these phenotypes can be reexamined under these conditions.*

We have included TEM images of the *P. aeruginosa* LESB58 wild-type as well as stringent response mutant after removal of cells from plates under surfing conditions (Figure S4), but emphasize that this cannot per se resolve mechanisms since we cannot perform TEM during surfing and since cells undergo substantial processing for TEM. TEM showed that stringent response mutant cells are phenotypically more similar to cells found at the edge of surfing cells. The text was modified accordingly (see response above). We thank the reviewer for these comments but, given these comments we do not believe that TEM of overexpressed *cueR* and *pqsH* would provide mechanistic insights.

*2) The transcriptome compares surfing wt and surfing stringent response. It is unclear how many of these changes are specific to the surfing condition or general to the stringent response regardless of growth condition. I would consider a control experiment, if not for the transcriptome at least for specific genes of interest with RT-PCR 1) wt and stringent response mutant in liquid growth. A comparison of swarming wt and mutant stringent response conditions would also be interesting since under both conditions the phenotype is completely abolished and can you identify surfing specific genes that altered in the stringent response under surfing compared with swarming*

Thank you for this feedback and idea. We have performed qRT-PCR experiments comparing the stringent response mutant to the wild type under swarming, swimming, and surfing conditions, of certain selected genes involved in flagella synthesis, chemotaxis, and quorum-sensing. Our data showed that flagella, chemotaxis, rhamnolipids, and quorum sensing are stringently-controlled during swarming motility, but not during surfing.

We have added the following to the results section:

Ln131-141: “Since surface motility adaptations (i.e., swim, swarm, surf) utilize flagella for colony propagation, we examined the expression of some genes involved in flagella synthesis, chemotaxis, and quorum-sensing using qRT-PCR (Table 1). RNA was isolated from *P. aeruginosa* LESB58 stringent response mutant cells and compared to wild-type cells from the motility edge. Under swarming conditions, downregulation in the Δ*relA*Δ*spoT* stringent response mutant was observed for the chemotaxis gene *cheY* by 3.8-fold, the two-component response regulator of flagella synthesis *fleR* by 3.5-fold, and the rhamnosyltransferase *rhlB* (which is involved in the synthesis of rhamnolipid, a surfactant required for swarming) by 5.8-fold. We also observed downregulation of the quorum-sensing regulators *lasR* and *rhlR* by 4.3- and 3.5-fold respectively. In contrast under surfing conditions, most of these genes were relatively upregulated or unaltered in expression in the double mutant (Table 1).”

**Table 1: Alterations in LESB58 Δ*relA*/Δ*spoT* mRNA transcripts under swarming, swimming, and surfing conditions.** The stringent response double mutant was compared to the edges of wild-type motility zones. All experiments were performed on KB agar plates.

| **Gene** | **Product Description** | Fold change in Δ*relA*/Δ*spoT* compared to WT | | |
| --- | --- | --- | --- | --- |
|  |  | **Surfing** | **Swarming** | **Swimming** |
| *fleQ* | [Transcriptional regulator FleQ](http://www.pseudomonas.com/feature/show?id=104960), a c-di-GMP responsive regulator that influences flagella synthesis | 1.7 | -1.0 | 1.1 |
| *fleR* | [Transcriptional response regulator FleR](http://www.pseudomonas.com/feature/show?id=1837827) that controls flagella synthesis | 1.1 | -3.5 | -1.4 |
| *cheY* | [Two-component response regulator CheY](http://www.pseudomonas.com/feature/show?id=1670530) that modulates chemotaxis | 1.7 | -3.8 | -1.5 |
| *rhlB* | [Rhamnosyltransferase chain B](http://www.pseudomonas.com/feature/show?id=109775) involved in the production of rhamnolipid surfactant required for swarming | 3.2 | -5.8 | -2.1 |
| *lasR* | [Transcriptional regulator LasR](http://www.pseudomonas.com/feature/show?id=105628) for the quorum sensing system based on N-3-oxo-dodecanoyl-L-homoserine lactone | 2.1 | -4.3 | 1.8 |
| *rhlR* | [Transcriptional regulator RhlR](http://www.pseudomonas.com/feature/show?id=109773) for the quorum sensing system based on N-butanoyl-L-homoserine lactone | 2.2 | -3.5 | -2.2 |
| *pqsH* | [FAD-dependent monooxygenase](http://www.pseudomonas.com/feature/show?id=1655804) involved in synthesis of the quorum sensing effector *Pseudomonas* Quinolone Signal (PQS) | -2.8 | -2.4 | -1.1 |
| *pqsR* | [Transcriptional regulator PqsR/MvfR](http://www.pseudomonas.com/feature/show?id=104770) modulates quorum sensing | 1.7 | 1.3 | -1.0 |
| *cueR* | [Cu(I)-responsive transcriptional regulator CueR](http://www.pseudomonas.com/feature/show?id=1673780) | -4.4 | -1.9 | -3.1 |

*3) The relationship between copper metabolism and other transcriptional changes. Quintana et al 2017 JBC show a major change in metabolism under copper stress. Could this explain some of the observed transcriptional changes. What happens to the other copper homeostatic pathways and if copper levels are manipulated within non toxic ranges. This is another opportunity for mechanism*

Thanks for this feedback. Tremblay et al [1] suggested that swarm center cells are under copper stress (although our own RNA-Seq data on swarming does not agree with this). In keeping with the reviewer’s comments, we looked further in our surfing data at the core copper regulon [2], however, only *queF* was significantly downregulated by 2.3-fold in the stringent response mutant. The role of QueF in copper homeostasis is rather minor and it is proposed to only fine-tune the activation of the copper homeostatic machinery [2]. Therefore, the copper homeostatic pathways did not appear to be under stringent control and do not favour a relationship between copper metabolism and metabolic changes observed in the stringent response mutant. Thus, we have not manipulated copper levels as this appears to be beyond the scope of the present report; but it would be of interest to further explore this area in future work.

1. Tremblay J, Deziel E. Gene expression in *Pseudomonas aeruginosa* swarming motility. BMC Genomics. 2010;11:587. Epub 2010/10/22. doi: 10.1186/1471-2164-11-587.
2. Quintana J, Novoa-Aponte L, Arguello JM. Copper homeostasis networks in the bacterium *Pseudomonas aeruginosa*. J Biol Chem. 2017;292(38):15691-704. doi: 10.1074/jbc.M117.804492.

***Reviewer #2:*** *There are inconsistencies in data from the different assays, which make their interpretation complex. These include: 1) The coloration of LESB58 in Fig 1A is differs from that of Fig 1B. Since the authors highlight differences in both pyoverdine and pyocyanin production for the relA/spot mutant as noteworthy, some validation or explanation is needed to better define the basic surfing assay. Similarly, is the strong pigmentation for the spoT-complement singularly noteworthy in comparison to the relA, pqsH, and cueR complements?*

These experiments were performed over the course of several months and images were taken with different cameras. The later pictures used a black and white camera only, and we therefore do not report on pigmentation. However, the change in coloration during surfing was observed only when the double mutant was complemented with *spoT*. While this is an interesting observation, we have not performed any follow-up experiments to investigate the role of SpoT in pigment production during surfing.

We have added the following statement:

Ln111-112: “This could be partially complemented with either the *spoT* or *relA* cloned genes, although the *spoT* complement was much more highly pigmented (Figure 1).”

The pyoverdine/pyocyanin experiment was based on the observation that LESB58 wild-type overnight cultures were blue/green in KB broth, while the stringent response mutant appeared yellowish (see Figure to the right). The obvious reduction in pyoverdine/pyocyanin was also consistent with the RNA-Seq data (under surfing conditions in the stringent response mutant) where most pyoverdine as well as pyocyanin biosynthesis genes were downregulated.

*2) The swimming assays shown in Figure 2A do not match swimming assays with which I am familiar. A swim phenotype should appear perfectly symmetrical as an expanding circle in the agar. These swim phenotypes are more circular than the swarm images above them, but have many irregularities. Further, the image shown suggests the growth is not uniform and may actually be on top of the agar. Given this, can the authors distinguish between swimming and surfing?*

While we agree that most swimming phenotypes are round and circular, we also have previously observed that swimming phenotypes can vary between bacteria [1] (and most likely also in isolates of the same species) and do not always follow this perfectly symmetrical pattern. Swimming with the LESB58 strain is more difficult to investigate since the phenotype after one day was not very clear in KB medium and incubation for two days often resulted in quite unusual swimming colony appearance. The representative swimming plates have now been moved to a separate Figure S1 and replaced with swimming in SCFM (where the phenotype is more clear), showing relatively weak swimming after 36 hours for LESB58 wild-type (with somewhat unusual patterns commented on in the text as mentioned above) and comparing this with swimming for strain PAO1 which is in fact fairly circular.

1. Sun E, Liu S, Hancock REW. Surfing motility: a conserved yet diverse adaptation among motile bacteria. J Bacteriol. 2018;200(23). Epub 2018/09/19. doi: 10.1128/JB.00394-18.

*The authors use of different experimental conditions also makes data interpretation difficult. Most assays were conducted using KB medium. However, pyocyanin assays were performed in SCFM, MIC assays were performed in MHB, and flow cell assays used dYT broth. Most importantly, if the authors seek to explain the relation between stringent response and surfing, mucin should be added to all experiments, as this is required for the surfing phenotype.*

We do not see this as a major issue since we were here confirming phenotypes observed by others in other *Pseudomonas* strains, rather than attempting to compare these different observations. Furthermore, previous studies from our lab [1] have shown that the surfing phenotype is primarily dependent on the presence of mucin and appears to quite independent of medium composition (cf. swarming). Although we have used different growth conditions for various experiments, we found that LESB58, the stringent response mutant, as well the complemented strains showed similar growth behavior under all tested conditions (KB, SCFM, dYT, MHB) (Figure S3, Table S1). Furthermore, the rationale for using SCFM over KB for pyocyanin determinations was that LESB58 did not produce significant amounts of pyocyanin in KB broth. We used MHB for MIC experiments since it is the standard recommended media according to the Clinical and Laboratory standards Institute. For biofilm formation, we have had greatest success in growing LESB58 in flow-cells when using dYT broth cf. any other media.

We agree that a proper comparison between the different experiments would require the same medium with and without mucin and agree with the reviewer that the experiments (other than surfing) distract from the main story; therefore decided to move these experiments to Supplementary information. Here, we specifically looked at the stringent response under surfing conditions and aimed to understand the mechanisms using RNA-Seq.

1. Sun E, Liu S, Hancock REW. Surfing motility: a conserved yet diverse adaptation among motile bacteria. J Bacteriol. 2018;200(23). Epub 2018/09/19. doi: 10.1128/JB.00394-18.

*The current manuscript oscillates between using the surfing phenotype as justification for stringent response experiments and using stringent response as justification for surfing experiments. Individually, many of these results are new and will be of interest to the field. However, the text presents a patchwork of data in search of a theme. Thus, the story requires more definition. If the point is to validate cueR and pqsH as important regulators of surfing, why not show how cueR and pqsH do (or do not) influence swarming, swimming, and antibiotic resistance?*

Thanks for this feedback. The LESB58 strain is not a commonly used type-strain; we wanted to first confirm and validate its phenotype with known phenotypes of the stringent response (from motility, growth, adherence and biofilm, to phenazine and siderophore production). However, we agree that this validation might distract from the major surfing story. We have modified the current version and moved the parts that did not address surfing motility into Supplementary. The main focus of the manuscript was to investigate the involvement of the stringent response in surfing motility and we found two novel downstream effectors. We have further introduced Table 1, where we performed qRT-PCR experiments comparing the stringent response mutant to the wild type under swarming, swimming, and surfing conditions, of certain selected genes including *cueR* and *pqsH*.

***Reviewer #3:*** *One important item I may have missed: Were the samples done in at least triplicates in the RNASeq analysis? The authors have to address this.*

Yes, all experiments were performed in triplicate (i.e. as 3 independent biological repeats). We added this to the methods section and included the PCA plot in Figure S10:

Ln406-407: “Three biological repeats with the highest RIN score were selected for further analysis.”

*I think the authors have to discuss the ramifications of the impact of 2 to 7.7 fold changes up or down in gene expression when doing transcriptomics in bacteria. It would be an opportunity to educate some of the readers that fold changes in bacteria remains a challenge. This is on page 9 line 2019 and applies even with qRT-PCR.*

Unfortunately there is no way of establishing the impact of specific changes in gene expression in that a very small change in a central metabolic gene or regulator might have a large impact relative to a much larger fold change in another gene. This is of course a major limitation of transcriptomic studies which provide potential rather than confirmed mechanisms. For this reason we have tried to limit the scope of our conclusions and implications.

***Part III – Minor Issues: Editorial and Data Presentation Modifications***

*Please use this section for editorial suggestions as well as relatively minor modifications of existing data that would enhance clarity.*

***Reviewer #1:*** *page 7 table 1 tobramycin resistance is listed for the complemented mutants which are on a gentamicin^R^ plasmid. These are both aminoglycosides and the plasmid level could contribute to tobramycin resistance.*

The mutants have been chromosomally complemented with a Tn7 transposon and while the gentamicin acetylase *aacC1* gene encoded on plasmid pUC18-mini-Tn7T-cassette was also transferred onto the chromosome, it does not modify or lead to resistance to tobramycin [1].

1. Poole K. Aminoglycoside resistance in *Pseudomonas aeruginosa*. Antimicrob Agents Chemother. 2005;49(2):479-87. Epub 2005/01/28. doi: 10.1128/AAC.49.2.479-487.2005.

*page 9: minor point: How was a defect in surfing defined (what percent of wt diameter, change in morphology etc)*

We have defined surfing deficiency as follows in the Methods section:

Ln337-339: “Surfing deficiency was identified either as an inability to spread past the point of inoculation, >70% reduced surface spreading, or a switch to in agar growth/motility which is characteristic of swimming rather than surfing which occurs on the surface.”

*In the discussion new data are introduced and discussed for the first time such as cyclic di-GMP regulon genes. I would move the data to the results and then discuss in discussion*

The data was moved accordingly.

*Figure 3. Would consider presenting the metabolic genes as a relational map to understand how the different changes and metabolic pathways relate to each other*

We thank the reviewer for the suggestion as it would indeed be helpful to visualize the data in this format. However, many of these relationships and components of metabolic pathways have not been extensively studied in surfing as it is a relatively novel form of motility. We feel the data as presented in Figure 2 (previous Fig 3) is sufficient to convey the metabolic changes within the scope of this manuscript. We believe an undertaking as significant as creating a metabolic map would be better suited for a more in-depth analysis of the impact of surfing motility and the stringent response on *Pseudomonas* metabolism. We hope that the continuing investigation in surfing motility will lead to a more comprehensive overview of how it contributes to key metabolic pathways.

***Reviewer #2:*** *1. Line 84, How is the CF lung more stressful than a healthy lung to P. aeruginosa where P. aeruginosa infections are less prevalent? Is increased mucin, itself, an inducer of stringent response? Or is it the use of antibiotics, as a means to treat infections, that stimulates stringent response?*

We have updated the according sentence and it reads now:

Ln83-85: The CF lung is considered a stressful environment due to increased oxidative stress [1], chronic activation of the innate immune system and consequent inflammation [2], chronic microbial infection and airway remodelling accompanied by mucous plugging.

1. Ntimbane T, Comte B, Mailhot G, Berthiaume Y, Poitout V, Prentki M, et al. Cystic fibrosis-related diabetes: from CFTR dysfunction to oxidative stress. Clin Biochem Rev. 2009;30(4):153-77. Epub 2009/12/17.
2. Ralhan A, Laval J, Lelis F, Ballbach M, Grund C, Hector A, et al. Current concepts and controversies in innate immunity of cystic fibrosis lung disease. J Innate Immun. 2016;8(6):531-40. Epub 2016/11/02. doi: 10.1159/000446840.

*2. Line 212, It would be useful to show the surfing phenotype of PA14 in the supplemental materials.*
The surfing phenotype of PA14 and the *cueR* as well as *pqsH* mutants (and complementation) were added as Figure S9.

***Reviewer #3:*** *Overall, all the critical experiments have been done. Careful analysis of the data and supplementary figures support this exciting manuscript. Overall, I see no major issues. I am looking forward to seeing this in print.*
