# Supplementary material for: Surfing motility is a complex adaptation dependent on the stringent stress response in Pseudomonas aeruginosa LESB58
Source: PLoS Pathog. 2020 Mar 24;16(3):e1008444. doi: 10.1371/journal.ppat.1008444 (PMC7122816; doi:10.1371/journal.ppat.1008444)
Supplement: Attachment — Submitted filename: Pletzer_PlosPathogens_2020_response2reviewers _round2_v2.docx [file ppat.1008444.s016.docx]

Dear Editor,

We wanted to thank you and all reviewers for their feedback. We have further responded in plain type for each issue in turn with the reviewer #1’s comments in italics. Modifications into the manuscript text are track changed in the marked copy of the manuscript.

Sincerely Yours

R.E.W. Hancock,

UBC Killam Professor and Canada Research Chair

*Responses to Questions*

*Please address the concerns of Reviewer 1 by clarifying the role of flagella in surfing motility. Also, in the revised manuscript, in text references to some of the supplemental data (e.g. Tables S1 and S2) have been removed. Please provide adequate text and references to this data in the manuscript or remove the supplemental information and renumber the existing tables/figures.*

We have addressed the concerns of Reviewer 1 below and updated the text/references to match the equivalent supplementary data.

***Reviewer #1:*** *The major experimental issues have been addressed. There is some confusion regarding the data that requires clarification prior to publication regarding the role of flagella regulation in the observed phenotype*

*Page 4 line 97 "In contrast to the studies of Vogt et al. [14] that suggested that ppGpp was not required for swimming in P. aeruginosa PAO1, as indeed we confirmed (Figure S2), we found that, in the strain LESB58, the stringent response mutant was further impaired in swimming after prolonged incubation time, but not completely defective" The picture in Figure S2 suggests to me it is completely defective. I tried to find a zone of swimming but could not. The authors then go on to state which is in direct contrast to both the figure and the previous statement. "There was no basic defect in flagella production due to any of these mutations, since we observed similar swimming zone sizes after 24 h for WT and all mutants in both the strains LESB58 and PAO1 (although the swimming colony morphology for strain LESB58 was somewhat unusual)"*

Swimming motility of the LESB58 strain is difficult to investigate since the strain has relatively lower motility cf. strain PAO1 and this phenotype is medium- and time-dependent. Originally, we used KB medium and took pictures after one day; but as one can see in the figure below, there was a swimming zone but it was rather small and there were no obvious differences between WT and mutant. Thus, based on this 24 hr observation, there was no indication that swimming was impaired in the stringent response mutant, as stated in the second sentence quoted in the reviewer’s comments.

Swimming - LESB58 in KB after 24h.

Even after 2 days (previous Figure S2), motility was rather modest cf. strain PAO1, but not in our opinion totally deficient in the stringent response mutant (NB this is point inoculation and you can clearly see in the panel above where the strains were inoculated). Thus, we do not agree that this represents “completely defective” motility as suggested by the reviewer. To clarify this, we have replaced the above panel into Figure S2 since issues such as the drying of the agar over 2 days make the 48 hour motility assay somewhat unreliable and clearly not convincing. The very confusing text was changed to read: “*Vogt et al. [14] demonstrated that ppGpp was not required for swimming in P. aeruginosa PAO1, as indeed we confirmed (Figure S2), and despite the rather poor swimming motility of strain LESB58, ppGpp did not appear to be required for the weak swimming motility observed for this strain either.*”

*In the results page 5 they state "We were unable to detect flagella in the spots formed by the relAspoT double mutant (Figure S4), although the large amount of extracellular material under these circumstances made observations difficult. This information along with the swimming figure has to make one consider whether flagella are present on the surface under these conditions. An up-regulation of flagella genes could be in response to protein degradation and lack of surface flagella. If there is identical swimming in aqueous solution amongst the mutants then this may indeed represent a novel and intriguing form of motility regulation depending on the environment regulated by the stringent response.*

Thanks for this valuable comment. As mentioned above, the wild type and stringent response mutant showed indeed identical swimming behaviour after 24 hours.

*Finally on page 13 of discussion this is a misleading sentence. "One cannot infer anything about surface expression and gene expression and while we did not observe more flagella on the stringent response mutant cells in TEM, a possible explanation could be that the up-regulation of the fle genes could lead to hyper-flagellated cells that might impair motility". In fact NO flagella were observed on the stringent response mutant cells. One can make no inference about surface protein and flagella structure expression based on gene expression alone. Although I agree that the extracellular material may make it difficult to see flagella, it is still a very sensitive technique. if the bacteria were hyper-flagellate it should be observable on TEM. There is no data to support this possible explanation and more data to support an opposite conclusion.*

We agree with the reviewer that the statement about gene expression and surface protein expression is very hypothetical and we cannot support that with any data at the moment. Therefore, we have deleted this sentence from the discussion and replaced it with ”This upregulation could be partially due to protein degradation and/or a deficiency of surface flagella”, as suggested by the reviewer.

*In summary the regulation of surface flagella and contribution to the observed phenotype is confusing and requires explanation.*

We hope we have addressed all the reviewers concerns appropriately. Thanks again for your valuable time and feedback.
